# Optimizing Use of Multistream Influenza Sentinel Surveillance Data

**DOI:** 10.3201/eid1407.080060

**Published:** 2008-07

**Authors:** Eric H. Y. Lau, Benjamin J. Cowling, Lai-Ming Ho, Gabriel M. Leung

**Affiliations:** * University of Hong Kong, Hong Kong Special Administrative Region, People’s Republic of China

**Keywords:** Sentinel surveillance, influenza, multivariate analysis, dispatch

## Abstract

We applied time-series methods to multivariate sentinel surveillance data recorded in Hong Kong during 1998–2007. Our study demonstrates that simultaneous monitoring of multiple streams of influenza surveillance data can improve the accuracy and timeliness of alerts compared with monitoring of aggregate data or of any single stream alone.

The use of separate data streams based on sentinel surveillance has long been an accepted approach to monitor community incidence and to enable timely detection of infectious disease outbreaks (*1**,**2*). Recently, more attention has been given to the combined analysis of multivariate sentinel data (*3*–*5*).

In this study we explored the possibility of improving the ability to more quickly detect peak periods of influenza activity in Hong Kong through simultaneous monitoring of multiple streams of sentinel surveillance data. Our findings have general implications in the choice of surveillance algorithms where multistream data are available.

## The Study

The local Department of Health publishes weekly reports (*6*) from a network of 50 private-sector sentinel general practitioners (GP) and 62 public-sector sentinel general outpatient clinics (GOPC) on the proportion of patients seeking treatment for influenza-like illness (ILI), defined as fever plus cough or sore throat (*7*). In this study, we used the GP and GOPC sentinel surveillance data in 9 annual influenza seasons from 1998–1999 to 2006–2007, stratified by 4 geographic regions in Hong Kong—Hong Kong Island, Kowloon, New Territories East, and New Territories West—resulting in 8 separate data streams (Figure).

**Figure:**
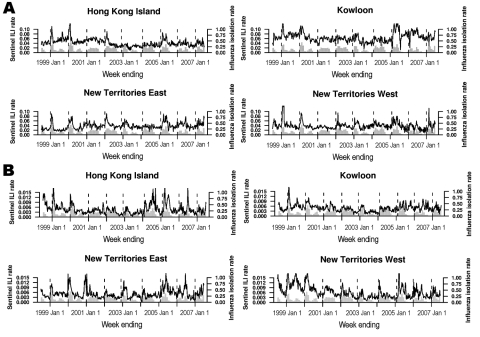
Nine annual cycles (unbroken lines) of general practitioner (A) and general outpatient clinic (B) geographic sentinel surveillance data from Hong Kong Island, Kowloon, New Territories East, and New Territories West, 1998–2007. The monthly proportions of laboratory samples testing positive for influenza isolates are overlaid as gray bars, and the beginning of each annual period of peak activity (inferred from the laboratory data) is marked with a vertical dotted line. ILI, influenza-like illness.

Each month a median of 1,555 specimens (interquartile range 1,140–2,740), primarily from hospitals, were sent to the Government Virus Unit of the Department of Health (*7*). We calculated the highest proportion of positive influenza isolations each season, and used these laboratory data to define the onset of each peak activity period when the proportion of positive influenza A or B isolates exceeded 30% of the maximum seasonal level (*7*).

Dynamic linear models (*8*) were used to generate alerts (Technical Appendix). We determined that an aberration had occurred when the current observation fell outside a forecast interval generated by the model. For methods based on monitoring of single data streams only, an aberration triggers an alert. For simultaneous monitoring of all 8 data streams, we monitored separate aberrations as above and generated alerts based on the first occurrence of any aberration (M1), 2 simultaneous aberrations (M2), the first occurrence of 3 simultaneous aberrations (M3), any 2 aberrations within a 2-week period (M4), and any 3 aberrations within a 2-week period (M5). In the multistream analyses, we compared alerts produced by univariate models, which effectively assumed independence between the data streams, and multivariate models, which allowed for correlation between the data streams (Technical Appendix).

Alerts were compared in terms of their sensitivity, specificity, and timeliness in detecting the onset of peak levels of influenza activity (*9*). We combined these metrics and estimated the area under the weighted receiver operating characteristic curve (AUWROC) as an overall measure of performance (*10*). The Table shows the highest AUWROC, for each method, from a predefined selection of parameter combinations and the sensitivity and timeliness at a fixed specificity of 95%. On the basis of aggregated data, we determined that alerts generated from the GOPC network achieved a higher AUWROC and better timeliness than those from the GP network. However, the best AUWROC from each of the data streams was produced by the GP New Territories East data, which outperformed the aggregate GP data. Conversely, for GOPC data, the performance of aggregate data was superior to that of any single data stream.

The Table also shows simultaneous monitoring results for all 8 geographic data streams from both GPs and GOPCs. For the univariate (independent) models for each data stream, methods based on simultaneous alerts perform well. The optimal methods were M2 and M3 with AUWROC of 0.89 and 0.90 and timeliness of 1.22 and 1.47 weeks, respectively, for a fixed specificity of 0.95. In general, univariate models performed better than multivariate models. Empirical correlation derived from one of the fitted multivariate models is shown in the Technical Appendix; correlation structures under other models were similar (data not shown).

Results were insensitive to the choice of parameters (Technical Appendix). The results also held when we varied the definition of the start of peak influenza activity between 10% and 50% of peak seasonal levels (Technical Appendix).

## Conclusions

A primary objective of sentinel surveillance is to provide sensitive, specific, and timely alerts at the beginning of increased disease activity (*11*). We evaluated the performance of multistream sentinel surveillance of ILI in detecting the onset of peak influenza activity.

Splitting sentinel data into separate geographic-based streams and monitoring all 8 streams for 2 or 3 simultaneous aberrations provided substantial improvements in AUWROC and also in timeliness for a fixed specificity when compared with monitoring aggregated data or any single data stream. We also used multivariate models with various alternative correlation structures between data streams, but use of these more complex models did not appear to improve performance (Table), possibly because correlation between streams vary year to year; the multivariate model is based on constant correlations (Technical Appendix). It is possible that other complex multivariate models may allow even greater improvement in performance; however, simultaneous monitoring of data streams may be more practical because univariate models may be applied in a spreadsheet (*7*).

**Table:** Performance of alerts generated by individual monitoring of aggregate data and separate data streams, and simultaneous monitoring of multiple data streams by using univariate and multivariate time series models, Hong Kong, 1998–2007*

Data	Univariate models				Multivariate models†		
	AUWROC	Sensitivity‡	Timeliness, wk‡		AUWROC	Sensitivity‡	Timeliness, wk‡
Aggregated data							
GP	0.78	1.00	2.41		–	–	–
GOPC	0.86	1.00	1.50		–	–	–
Single stream							
GP							
HK	0.75	1.00	2.36		0.73	0.87	2.64
KL	0.66	1.00	2.71		0.62	0.88	3.06
NTE	0.89	1.00	2.00		0.76	0.90	2.04
NTW	0.80	1.00	2.07		0.80	0.91	2.24
GOPC							
HK	0.79	1.00	2.21		0.71	0.89	2.42
KL	0.78	1.00	2.46		0.62	0.96	3.15
NTE	0.79	0.95	2.22		0.79	0.96	2.26
NTW	0.73	1.00	2.55		0.72	1.00	2.52
Multiple streams							
M1: First aberration	0.84	1.00	1.57		0.86	1.00	1.66
M2: 2 simultaneous aberrations	0.89	1.00	1.22		0.82	1.00	1.77
M3: 3 simultaneous aberrations	0.90	1.00	1.47		0.80	1.00	1.70
M4: Any 2 aberrations in 2 wk	0.81	1.00	2.63		0.72	1.00	2.43
M5: Any 2 aberrations in 2 wk	0.83	1.00	2.44		0.77	1.00	2.11

*AUWROC, area under the weighted receiver operating characteristic curve; GP, general practitioner; GOPC, general outpatient clinic; HK, Hong Kong Island; KL, Kowloon; NTE, New Territories East; NTW, New Territories West. †See Technical Appendix for more detailed description of the multivariate model. ‡At a fixed specificity of 0.95.

Although the relative performance of GP and GOPC sentinels may not be directly generalizable to other settings with differences in infectious disease dynamics and healthcare systems, the implications for data collection are nevertheless relevant. Inclusion of data streams should be based on their value to the overall surveillance system, rather than independent performance. For example, simultaneous monitoring of data streams where some have lower specificity and others have higher specificity could still improve overall timeliness.

Specifically regarding Hong Kong, it is unclear why alerts from the private GP network have better timeliness than those from the public GOPC network. Although we note that both networks have different catchment populations, the GOPC network typically serves elderly and lower income groups (*12*), whereas influenza would be more likely to affect children at the start of the influenza season (*13*). Differences between geographic regions could be real, when disease progresses from 1 region to another (*14*); however, this circumstance is unlikely in Hong Kong, an area of only 1,000 km^2^, where a high degree of mixing occurs among a population of 7 million persons. Geographic heterogeneity could also be explained by differential socioeconomics and demographics between different regions, associated differences in access to healthcare and health-seeking behavior issues, or small area variations in reporting behavior among the sentinel practices.

A potential caveat of our analysis is the small number of annual cycles of sentinel data available for study. However, until recently, few subtropical or tropical regions had begun influenza sentinel surveillance. Another limitation is the absence of a generally agreed-upon standard in defining a peak influenza season. In our analysis, the start of peak activity was defined as laboratory isolation rates exceeding 30% of the annual level; however, we found that our results were not sensitive to other reasonable thresholds. In addition, we compared methods with only a few chosen parameter combinations; sensitivity analyses showed that the results were not sensitive to the smoothing parameter or the specification of correlations between streams. Finally, alerts generated by other more complicated combinations of aberrations might provide further enhancements. However, the value of simultaneously monitoring separate data streams (*15*) has already been demonstrated by the simple combinations chosen here.

## Supplementary Material

Technical Appendix Optimizing Use of Multistream Influenza Sentinel Surveillance Data
