# Supplementary material for: Optimizing Use of Multistream Influenza Sentinel Surveillance Data
Source: Emerg Infect Dis. 2008 Jul;14(7):1154–7. doi: 10.3201/eid1407.080060 (PMC2600317; doi:10.3201/eid1407.080060)
Supplement: Technical Appendix — Optimizing Use of Multistream Influenza Sentinel Surveillance Data [file 08-0060_Techapp-s1.pdf]

Optimizing Use of Multistream Influenza Sentinel Surveillance Data

Technical Appendix

The dynamic linear model (*8*) is a form of state space model for time series data. In these models, observed data $\boldsymbol{y}_t$ (representing a vector of data at time t) are assumed to be related to some underlying unobserved sequential process, or system. Allowing for this underlying system where subsequent (unobserved) system values $\boldsymbol{\theta}_t$ are highly correlated, the consecutive observations $\boldsymbol{y}_t$ are assumed to be conditionally independent. With a Bayesian approach under this structure, multivariate normal distribution theory allows simple parameter estimation. Formally, the model is described by the following equations:

$$\boldsymbol{y}_t = \boldsymbol{\theta}_t + \boldsymbol{v}_t \quad \text{where } \boldsymbol{v}_t \sim N(0, \boldsymbol{V}), \text{ and}$$

$$\boldsymbol{\theta}_t = \boldsymbol{\theta}_{t-1} + \boldsymbol{w}_t \quad \text{where } \boldsymbol{w}_t \sim N(0, \boldsymbol{\Sigma}).$$

The observation error $\boldsymbol{v}_t$ and the evolution error $\boldsymbol{w}_t$ are internally and mutually independent, and the model parameters and the observation variance matrix $\boldsymbol{V}$ may be estimated from the data by using a Kalman filter (*7,8*), i.e., without requiring maximization of a likelihood function. The evolution variance matrix can be prespecified. In the case of univariate data (i.e., separate data streams), the above vectors and matrices simplify to scalars.

In our specific application where we model 8 data streams i.e., from 2 separate surveillance networks each covering 4 geographic areas, 3 types of correlation naturally arise, namely within-source correlation, within-region correlation, and residual correlation. We specified the evolution variance-covariance matrix $\boldsymbol{\Sigma}$ in the following form:

$$\boldsymbol{\Sigma} = \sigma^2 \begin{pmatrix} W & A \\ A^T & W \end{pmatrix}$$

with $$\boldsymbol{W} = \sigma^2 \begin{pmatrix} 1 & \rho_W & \rho_W & \rho_W \\ \rho_W & 1 & \rho_W & \rho_W \\ \rho_W & \rho_W & 1 & \rho_W \\ \rho_W & \rho_W & \rho_W & 1 \end{pmatrix}$$

and $\boldsymbol{A} = \begin{pmatrix} \rho_I & \rho_R & \rho_R & \rho_R \\ \rho_R & \rho_I & \rho_R & \rho_R \\ \rho_R & \rho_R & \rho_I & \rho_R \\ \rho_R & \rho_R & \rho_R & \rho_I \end{pmatrix}$

Equal correlation was assumed between geographic regions for data streams from the same surveillance network, specified by the parameter ρ_w for the off-diagonal elements in $\boldsymbol{W}$. The parameter ρ_I denotes the correlation within region but under different surveillance networks, and ρ_R denotes residual correlation between data streams in different regions and sources. Several sets of correlation parameters were prespecified to assume different levels of dependence between data streams, assuming that $\rho_W > \rho_I > \rho_R$. We applied the multivariate model using sets of (ρ_W, ρ_I, ρ_R) = (0, 0, 0), (0.3, 0.1, 0), (0.5, 0.2, 0.1), (0.8, 0.2, 0.1) and (0.8, 0.4, 0.3), denoted by $\boldsymbol{\Sigma}_i$, i =1, 2, …, 5, respectively, to represent a spectrum of assumptions in the correlation structure between data streams (from no or low inter and intradependence within source or region to high dependence). The overall evolution variance σ^2 is also varied, in combination with different dependence structure, as 0.025, 0.050, 0.075 and 0.100 to represent high- to-low smoothness in the assumed change in influenza prevalence.

Having fitted the model for data at times 0,…, *t-1*, a one-step-ahead forecast interval may be estimated for time *t*. Varying the coverage of the forecast interval provides a range of values for the 3 metrics of sensitivity, specificity, and timeliness. Increasing the width of the forecast interval will result in fewer aberrations (an aberration occurs when an observed value exceeds the forecast interval) and, in general, will increase the specificity but decrease the sensitivity and timeliness.

For simultaneous monitoring of separate data streams based on univariate models, a dynamic linear model was fitted separately to each data stream and individual aberrations were combined to generate overall alerts. This approach is very similar (but not quite identical) to assuming $\rho_W = \rho_I = \rho_R = 0$ in a multivariate model.

Estimating the AUWROC

The area under the weighted receiver operating characteristic curve (AUWROC) is a combined metric of sensitivity, specificity, and timeliness. First, the time saved (TS) is calculated as 1 minus the ratio of timeliness to a predefined maximal delay of alerts. In this scenario of influenza surveillance, considering the short generation time of the disease, an

early warning should be issued ideally within ≈4 weeks of the start of the peak season; therefore, we used 4 weeks as the maximal delay of alerts. By setting different thresholds for each method to obtain sets of sensitivity, specificity, and timeliness, a weighted ROC curve was then plotted by TS × sensitivity against the false positive rate (1 – specificity); the area under the curve is the AUWROC.

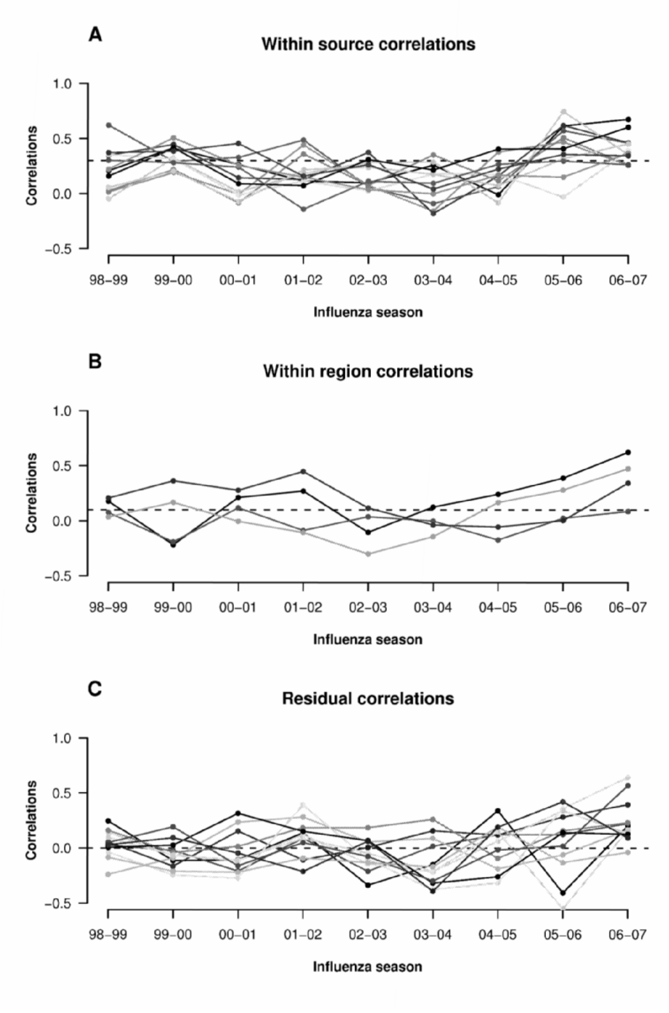

Figure. Empirical evolution correlations obtained from the dynamic linear model with parameters σ = 0.75, (ρ_W, ρ_I, ρ_R) = (0.3, 0.1, 0) over 9 influenza seasons including within-source correlations (A) within-region correlations (B), and residual correlations (C) with the horizontal lines showing the respective assumed correlations in the model.

Table 1. Sensitivity analyses of the performance of alerts generated by simultaneous monitoring of multiple data streams by using univariate and multivariate time series models*

	AUWROC						
	Parameters	Univariate	Multivariate models (ρ_W, ρ_I, ρ_R)				
Method	σ^2	models	(0, 0, 0)	(0.3, 0.1, 0)	(0.5, 0.2, 0.1)	(0.8, 0.2, 0.1)	(0.8, 0.4, 0.3)
M1: first aberration	0.025	0.84	0.84	0.82	0.81	0.81	0.81
	0.050	0.83	0.84	0.83	0.81	0.81	0.81
	0.075	**0.84**	0.85	0.83	0.81	0.82	0.81
	0.100	0.84	**0.86**	0.83	0.82	0.83	0.83
M2: 2 simultaneous aberrations	0.025	0.87	0.75	0.76	0.76	0.79	0.80
	0.050	0.88	0.78	0.80	0.79	0.80	0.80
	0.075	**0.89**	0.79	0.80	0.81	0.81	0.80
	0.100	**0.89**	**0.82**	0.80	0.81	0.81	0.81
M3: 3 simultaneous aberrations	0.025	0.89	0.74	0.75	0.74	0.74	0.77
	0.050	0.89	0.76	0.75	0.74	0.75	0.77
	0.075	0.89	0.75	0.78	0.76	0.77	0.77
	0.100	**0.90**	0.77	**0.80**	0.77	0.77	0.80
M4: any 2 aberrations in 2 wk	0.025	0.79	0.70	0.67	0.68	0.71	0.71
	0.050	0.78	0.68	0.70	0.70	0.70	0.71
	0.075	0.79	0.71	0.71	0.69	**0.72**	0.71
	0.100	**0.81**	0.71	0.67	0.70	0.70	0.71
M5: any 3 aberrations in 2 wk	0.025	**0.83**	0.67	0.70	0.69	**0.77**	0.73
	0.050	0.83	0.67	0.73	0.70	0.71	0.70
	0.075	0.82	0.69	0.72	0.71	0.71	0.70
	0.100	0.83	0.72	0.69	0.74	0.70	0.70

*AUWROC, area under weighted receiver operating characteristic curve. Optimal parameter combinations for each method under univariate and multivariate models are shown in **boldface**.

Table 2. AUWROC of different methods with the start of influenza season defined as influenza isolation rates exceeding 10%, 30%, or 50% of the seasonal peak level*

	AUWROC/threshold					
	10%		30%		50%	
Data	Univariate	Multivariate	Univariate	Multivariate	Univariate	Multivariate
Aggregated data						
GP	0.79	–	0.78	–	0.84	–
GOPC	0.76	–	0.86	–	0.85	–
Single stream						
GP						
HK	0.60	0.62	0.75	0.73	0.77	0.81
KL	0.75	0.62	0.66	0.62	0.76	0.75
NTE	0.78	0.69	0.89	0.76	0.91	0.83
NTW	0.82	0.77	0.80	0.80	0.87	0.85
GOPC	0.61	0.64	0.79	0.71	0.88	0.80
HK						
KL	0.68	0.61	0.78	0.62	0.85	0.76
NTE	0.72	0.70	0.79	0.79	0.79	0.74
NTW	0.80	0.70	0.73	0.72	0.83	0.80
Multiple streams						
M1: first aberration	0.83	0.84	0.84	0.86	0.89	0.88
M2: 2 simultaneous aberrations	0.77	0.77	0.89	0.82	0.90	0.88
M3: 3 simultaneous aberrations	0.77	0.72	0.90	0.80	0.92	0.90
M4: Any 2 aberrations in 2 wks	0.85	0.77	0.81	0.72	0.84	0.82
M5: Any 3 aberrations in 2 wks	0.83	0.75	0.83	0.77	0.87	0.86

*AUWROC, area under weighted receiver operating characteristic curve; GP, general practitioner; –, not applicable; GOPC, general outpatient clinic; HK, Hong Kong Island; KL, Kowloon; NTE, New Territories East; NTW, New Territories West.
